# Metabolomic Approaches to Investigate the Effect of Metformin: An Overview

**DOI:** 10.3390/ijms221910275

**Published:** 2021-09-24

**Authors:** Hyun Woo Kim

**Affiliations:** Center for Marine Biotechnology and Biomedicine, Scripps Institution of Oceanography, University of California San Diego, La Jolla, CA 92093, USA; hwkim@ucsd.edu; Tel.: +1-(858)-333-2648

**Keywords:** metformin, metabolomics, T2DM, metabolome, BCAAs, TCA cycle

## Abstract

Metformin is the first-line antidiabetic drug that is widely used in the treatment of type 2 diabetes mellitus (T2DM). Even though the various therapeutic potential of metformin treatment has been reported, as well as the improvement of insulin sensitivity and glucose homeostasis, the mechanisms underlying those benefits are still not fully understood. In order to explain the beneficial effects on metformin treatment, various metabolomics analyses have been applied to investigate the metabolic alterations in response to metformin treatment, and significant systemic metabolome changes were observed in biofluid, tissues, and cells. In this review, we compare the latest metabolomic research including clinical trials, animal models, and in vitro studies comprehensively to understand the overall changes of metabolome on metformin treatment.

## 1. Introduction

Metformin (N,N-dimethyl biguanide) is a first-line anti-hyperglycemic medication for the treatment of type 2 diabetes mellitus (T2DM), particularly in overweight or obese patients, by lowering the glucose levels and improving insulin sensitivity [1]. Clinically, metformin treatment reduces body weight and adiposity, and positively affects glucose homeostasis in T2DM patients [2].

**Figure 1:**
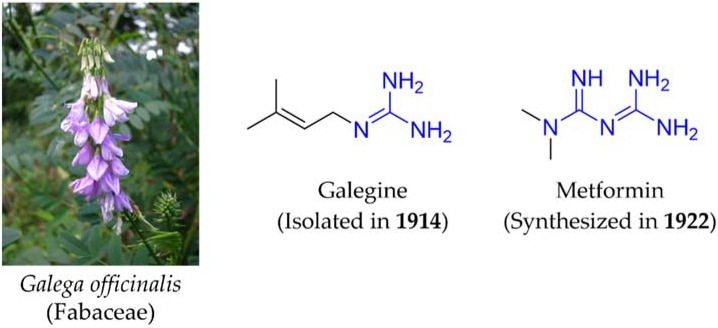
Galega officinalis (Goat’s rue), galegine, and metformin.

The discovery of metformin is derived from galegine, a natural product isolated from the medicinal plant *Galega officinalis* (Goat’s rue), which has been traditionally used in Europe [3]. Galegine, a guanidine alkaloid containing an isoamylene, is known to enhance glucose uptake and inhibition of acetyl-CoA carboxylase and is thus expected to contribute to the weight-reducing effect [4]. However, *G. officinalis* is toxic to animals, shown through accidental livestock poisoning in the field as well as laboratory studies. Additionally, galegine is believed to be the major toxin of *G*. *officinalis* and causes blood pressure lowering, paralysis, and death in sheep [5]. Metformin is structurally modified from galegine containing a biguanide moiety (Figure 1). Interestingly, this small modification decreased the toxicity, but still keeps the glucose lowering effect [6]. Pharmacokinetically, metformin does not undergo hepatic metabolism, so it is considered safe from a hepatic standpoint, and metformin-induced hepatotoxicity is very rare, with less than 20 cases having been described over several decades [7]. Various clinical studies also supported the safety and tolerability of metformin treatment [8,9,10]. Thus, metformin has been used for almost 70 years to treat T2DM patients with its safety and clinical usefulness.

Chemically, metformin is a hydrophilic base, which exists as a cationic species at a physiological pH. Regarding this poor lipophilicity, passive diffusion of metformin through cell membranes is limited; thus, its uptake and distribution crucially rely on the organic cation transporters (OCTs) which are expressed at significant levels in metformin target tissues such as liver, muscle, and adipose tissue. Deficiency or inhibition of the OCTs impairs the uptake of metformin in the small intestine and the hepatic uptake and elimination and attenuated antiglycemic effect by metformin treatment [11,12,13,14].

The mechanism of action of metformin is complex and still not fully understood [15]. Traditionally, it is accepted that the anti-hypoglycemic activity of metformin is involved in the suppression of hepatic gluconeogenesis [16,17,18]. In the liver, metformin regulates mitochondrial respiration by inhibiting the mitochondrial respiratory-chain complex 1 and activating the AMP-activated protein kinase (AMPK) in hepatic gluconeogenesis [19,20]. Metformin also increases AMPK activity and insulin-stimulated glucose transport in skeletal muscle [21,22]. However, contrast results were also reported from several studies where hepatic gluconeogenesis increased in nondiabetic patients and recent-onset T2DM patients, indicating the mechanism of action of metformin may be extra-hepatic [23,24]. Recently, the intestines are another target of metformin by increasing anaerobic glucose metabolism in enterocytes to reduce glucose absorption and increase lactate delivery to the liver [25]. Gut microbiota, such as *Akkermansia muciniphila*, *Escherichia* spp., or *Lactobacillus,* and their composition has been altered by metformin as well [26,27].

Even though the mechanisms of drugs have been revealed, with a revolution in biomedical research that resulted in remarkable therapeutic advances in drug development, various responses from patients to a drug treatment are still challenging [28]. For example, metformin treatment was considered to have failed to control glucose levels in approximately 15% of patients [29,30]. Age, sex, environment, nutrition, microbiome, and other internal/external factors can impact the drug response of individual [31,32,33].

From the perspective of precision medicine, interpretation and prediction of the biological phenotype including drug responses or side-effects are becoming an important concern in medication [34]. Discovery of the biomarkers and development of diagnostics for the prediction require efficient computational tools and a substantial number of samples. To achieve those, metabolomics research has been concerned. Metabolomics is a systematic study of the unique chemical fingerprints based on the high-throughput identification, quantification, and characterization of the small molecule metabolites in the metabolome, which is the complete complement of all small molecules found in a specific cell, tissue, organ, or organism [35].

**Figure 2:**
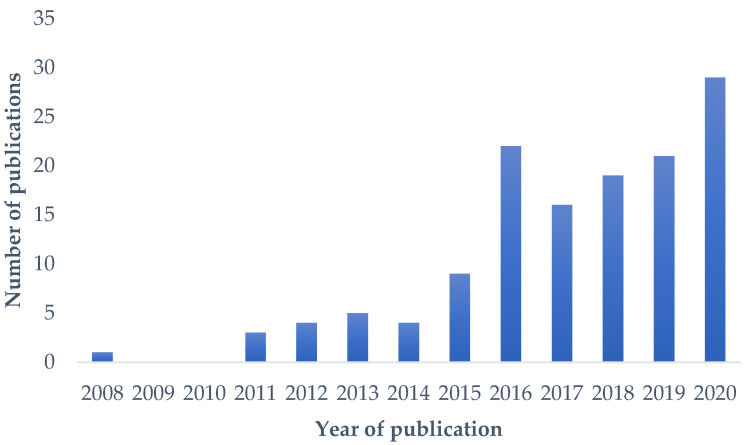
Number of papers (Web of Science^®^) published per year on the topics of metformin and metabolomics.

Here, we discuss the recent research understanding the effect of metformin and their metabolism, mainly focusing on metabolomics approaches (Figure 2). In addition, we will review the potential biomarkers of metformin treatment and the relationship between metabolites.

## 2. Metabolomics

Metabolomics is the large-scale identification and quantification study of all targeted metabolites within cells, biofluids, tissues, or organisms under specific conditions or diseases [35]. These metabolites are small molecules within a mass range of 50 to 1500 Daltons, and their interaction within a biological system are known as the metabolome, which is defined as the collection of the metabolites as well. Endogenous metabolites such as sugars, lipids, amino acids, fatty acids, nucleic acids, or organic acids are typically produced from the metabolism and considered as targets of metabolomics study. In the central dogma of biology, metabolites are the downstream summation of DNA, RNA, and proteins, which means any change in these molecules affects the metabolomes, as well as the changes in environment such as diet, exercise, mental stress, and disease [36]. In other words, metabolic profiling is to take a snapshot of the physiologic status of a biological system, and the metabolome changes inferred the relation between the genome (DNA), transcriptome (RNA), proteome (proteins) and biological phenotypes such as drug response or side effects [37].

Various analytical platforms and technological advances have allowed researchers to detect, identify and quantify thousands of metabolites from complex biofluids. Mass spectrometry (MS) and nuclear magnetic resonance spectroscopy (NMR) are the most common analytic platforms with complementary usefulness in various metabolomic studies [38,39]. MS gives information about molecular weight while also identifying and quantifying the compounds under analysis. MS is frequently coupled to liquid chromatography (LC) to establish an LC-MS platform, or gas chromatography (GC) to establish GC-MS, which are powerful analytical techniques that combine the physical separation of chromatography with highly sensitive and selective mass analysis of MS. This hyphenated platform enables the simultaneous and integrated analysis of hundreds of metabolites in complex samples with high sensitivity and selectivity. A liquid chromatography–tandem mass spectrometry (LC-MS/MS) platform, where triple quadrupole mass spectrometers (QQQ) or quadrupole time-of-flight (QTOF) is integrated, is the most commonly used platform. However, MS cannot detect all metabolites, as some metabolites do not ionize with certain ionization methods. So, the number and class of metabolites that can be detected by MS depends on the choice of ionization mode. Compared with MS, NMR spectroscopy is a quantitative and highly reproducible analytical platform providing information of metabolites in almost all biofluids, tissues, cell extracts, or even live cells, as well as being a non-destructive analytical technique. The types of molecules do not influence the NMR results as well. NMR-based metabolomics gives useful information about the presence/absence or the number of metabolites in a biological system and enables monitoring the flow of compounds through metabolic pathways. For instance, around one hundred metabolites in human urine were identified and quantified by analyzing ^1^H NMR-based metabolic profiles, providing the effects of feeding and body-weight loss in energy metabolism [40]. Two-dimensional NMR experiments such as ^1^H-^1^H correlation spectroscopy (COSY), ^1^H-^13^C heteronuclear single quantum coherence (HSQC), and total correlation spectroscopy (TOCSY) are used to identified complex biofluid mixture as well. The low sensitivity is a weakness of NMR but can be improved with higher field strength, cryoprobe, and dynamic nuclear polarization techniques.

Metabolomics study is typically classified into two categories: targeted and untargeted metabolomics [41]. In targeted metabolomics, clearly defined and selected compounds are analyzed and compared from different sample groups. This approach involves the measurement of identified and chemically defined compounds, which is related to the metabolic pathway or hypothesis. Therefore, unknown or non-targeted metabolites are not considered. On the other hand, untargeted metabolomics mainly focuses on the global consideration of both known and unknown metabolites for comprehensive analysis to detect and figure out an alteration from different conditions in order to identify and relatively quantify the metabolites with different contributions in terms of classification, for example, healthy and diseased. Novel metabolism or metabolic pathways can be revealed from the changes in metabolic profiles. However, identification of significant peaks remains a challenge, complicating in-depth mechanistic or biochemical understanding.

In the pharmaceutical science field, pharmacometabolomics has arisen from the metabolomics research field to achieve enhanced and systemic understanding of mechanisms for drug or xenobiotic effects and improve the ability to predict individual variation in drug response phenotypes based on using both baseline metabolic profiles prior to treatment and also the effects of drug treatment overtime [42]. Clinically, pharmacometabolomics is anticipated to enable precision medicine given by monitoring drug response and treatment outcomes, finding novel response pathways or biomarkers, and combining with pharmacogenomics for understanding drug affects [43].

## 3. Plasma Metabolome and Metformin Treatment

The definition of plasma is the clear, straw-colored liquid portion of the blood that remains after red blood cells, white blood cells, platelets and other cellular components are removed. It is the single largest component of blood, comprising over half of the volume, and various classes of metabolites with different chemical properties are in the plasma. Changes in plasma biomarker concentrations are not necessarily related to specific organs or tissues and can be the product of a systemic response [44]. Thus, plasma metabolomic profiles are widely used in various disease studies such as T2DM [45], chronic kidney disease (CKD) [46], cancer [47], malaria [48], obesity [49], depression [50], hypertension [51], and Alzheimer’s disease [52], providing new insights into systemic mechanisms underlying the pathophysiology of the diseases. Currently, around four thousand metabolites have been detected or quantified, and twenty thousand metabolites are expected to be present in human blood [53].

### 3.1. Effect of Metformin Treatment in Non-Diabetic Condition

Metformin treatment in non-diabetic status influenced the various metabolite levels in blood. Cai et al. [54]. performed a one-self control study with a metformin tablet of 500 mg twice daily and compared the plasma metabolite profiling in 20 healthy volunteers before and after metformin treatment for 7 days, showing significant difference in endogenous metabolite profiles using LC-MS. The levels of four lysophosphatidylcholines (LPCs) including 16:0 LPC, 18:0 LPC, 18:1 LPC, and 18:2 LPC of metformin treatment groups were obviously decreased compared with the control group, which indicated metformin regulates LPC levels in plasma and improves lipid metabolism. Dahabiyeh et al. [55] performed an MS-based untargeted metabolomics approach to investigate the metabolic changes associated with the administration of a single dose of metformin (500 mg) in 26 healthy subjects. Among the endogenous metabolites, 5-aminopentanoic acid, propionic acid, hydroxymethyl uracil, and ethyl phenyl sulphate significantly increased, and metabolites involved in arachidonic and linoleic acid metabolisms, glycerophospholipids, and eicosanoids decreased. Their results pointed out that the roles of metformin could be associated with fundamental biochemical processes such as lipid network signaling, energy homeostasis, DNA repairing, and gut microbiota.

Branched-chain amino acids (BCAAs), namely isoleucine, leucine, and valine and aromatic amino acids (AAs) showed a strong association with future diabetes as well as obesity and serum insulin level [56,57,58]. Preiss et al. [59] focused the metabolic shift on circulating amino acids in response to metformin treatment (1700 mg, daily) in 173 individuals without T2DM, but with coronary disease from the Carotid Atherosclerosis: Metformin for Insulin Resistance (CAMERA) randomized controlled trial study, and observed the increase in alanine and histidine levels, the decrease in tyrosine and phenylalanine levels, and no changes in BCAA levels in plasma by ^1^H NMR data analysis, but those changes of aromatic amino acids and alanine levels were not related to the body weight and insulin sensitivity. Walford et al. [60] also reported no changes in BCAA levels and the decrease in tyrosine level in the plasma from 30 insulin-sensitive subjects with 2 days of metformin treatment (500 mg, twice daily) by LC-MS analysis with a hydrophilic interaction liquid chromatography. Rotroff et al. [61] performed LC-MS-based non-targeted pharmacometabolomics analysis to investigate the effect of metformin (1000 mg for the first day and 850mg for the second day) in 33 non-diabetic African Americans and reported several metabolic alterations with indole-3-acetate, 4-hydroxyproline, and 2-Hydroxybutanoic acid, revealing that metformin was associated with the urea cycle and purine metabolism. The significant decrease in ornithine and citrulline levels in plasma indicated that metformin administration was related to the mitochondrial complex I, the primary target of metformin. Interestingly, 2-Hydroxybutanoic acid was positively correlated with fasting glucose levels as well as glucose levels following oral glucose tolerance test (OGTT) after metformin treatment in their study. However, there were no significant changes in BCAA levels. Gormsen et al. [24] carried out a randomized, placebo-controlled trial in 24 subjects with recent-onset T2DM and 12 non-diabetic individuals with metformin administration (1000 mg twice daily) for 90 days. In the study, the metabolic profile in the plasma was analyzed by non-targeted metabolomics research, and 882 named biochemicals were identified by using LC-MS and GC-MS. In their study, no significant alteration in BCAA levels was observed, and a decrease in tyrosine was shown.

Table 1 shows the significantly altered plasma metabolites and related metabolism in response to metformin treatment, which is reported from multiple studies. Most metabolites showed similar trends in different studies without hippuric acid, hypoxanthine, and arginine. This difference might be from different environments, nutrients, or other internal/external factors.

### 3.2. Effect of Metformin Treatment in Insuline-Resistant Condition

Various studies reported the effect of metformin on the plasma metabolome profiles associated with obesity and T2DM cases as well as blood glucose level (Table 2). Gormsen el al. [24] reported that metformin treatment (1000 mg twice daily) altered the metabolic profiles and found that fasting plasma glucose level decreased in only the metformin-treated T2DM group, and 1,5-anhydroglucitol (1,5-AG) was associated with a glucose-lowering effect in response to metformin administration. Huo et al. [62] compared the biochemical changes in the serum of 35 T2DM patients with/without the treatment of metformin (the dose was not described) for 3 months using ^1^H NMR and UPLC/MS analysis and observed the elevation of trimethylamine-*N*-oxide (TMAO), 3-hydroxybutyrate (3-HB), and tryptophan, and the reduction in acetoacetate, unsaturated lipids, and LPCs (C16:0 LPC, C18:0 LPC, and C18:2 LPC). Among the altered metabolites, TMAO is related to the gut microbiota metabolism, so its alteration indicated an intestinal bacteria regulation function of metformin. Xu et al. [63] analyzed both metabolomic and genomic data of the population-based KORA cohort and studied the effects of metformin on metabolite profiles and LDL cholesterol in T2DM patients to discover metformin treatment-associated metabolites (the dose was not described). Among the 130 metabolites in fasting serum, three metabolites, including three phosphatidylcholine acyl-alkyls (PC ae C36:4, PC ae C38:5, and PC ae C38:6), which are composed of at least one polyunsaturated fatty acid (PUFA) remained significantly different in the comparison between metformin and control groups. Those metabolites are involved in the AMPK pathway associated with *FADS1* and *FADS2* genes. Adam et al. [64] carried out a further cohort study with LC-MS-based non-targeted metabolite profile study, reporting that a significant lower plasma citrulline relative concentration was observed in the metformin treatment group. Additionally, ornithine, urea, and arginine concentrations were lowered in human serum. This phenomenon was also observed in the diabetic mice model following daily, subchronic metformin treatment (300 mg/kg/day) compared with the control mice. Lower citrulline results were also observed in murine skeletal muscle and adipose tissue, but not in the liver. Breier et al. [65] also reported the immediate and sustained reduction in the serum citrulline level after initiation of metformin (500–1000 mg daily for the first dose, 1000–2000 mg daily for 4 to 6 weeks) in T2DM patients by using LC-MS, but the serum steroid profile was not altered. Another randomized, double-blind, placebo, controlled study from Irving et al. [66] reported the lowering of citrulline and arginine concentration in the pioglitazone-metformin combination therapy (45 mg of pioglitazone daily plus 1000 mg of metformin twice daily). Those results announced that the urea cycle could be downregulated by metformin.

Metformin treatment also affects BCAAs, including leucine, isoleucine, and valine, and AAs, including tryptophan, tyrosine, phenylalanine, and histidine levels, in insulin-resistant conditions. The metabolism of certain amino acids is related to insulin-resistant states, so glucose-lowering medications could alter amino acid concentrations as a downstream consequence. In the insulin-resistant cases, BCAAs were significantly affected by metformin, but the response was different in humans and mice. Safai et al. [68] analyzed 87 plasma metabolites in 370 participants with T2DM from a randomized (1:1 of metformin or placebo treatment) study using LC-MS, and reported that higher levels of leucine/isoleucine and lower levels of carnitine, tyrosine, and valine were observed in T2DM participants treated with metformin (1000 mg twice daily) over 18 months, but there was no correlation between the markers and HbA_1c_ levels. Huhtala et al. [67] measured the serum concentration of alanine, glutamine, glycine, isoleucine, leucine, valine, histidine, phenylalanine, and tyrosine along with glucose and lactate in women with gestational diabetes mellitus treated with metformin (500 mg daily and increased to 2000 mg daily if needed) or insulin by analyzing ^1^H NMR spectroscopy data and observed the rise in alanine, isoleucine, and lactate in the metformin group compared to the insulin group. Walford et al. [60] reported acute increases of BCAAs and AAs following metformin treatment in insulin-resistant subjects. On the other hand, opposite results were reported in an LC-MS-based T2DM mice model study with metformin treatment (250 mg/kg/day), in which carnitine (butyrylcarnitine, acetylcarnitine, and acylcarnitine C18:1) levels increased and isoleucine concentration decreased [69]. Sonnet et al. [70] also observed that metformin treatment (0.1% *w*/*w* ad libitum) reduced serum BCAA levels in the murine model of maple syrup urine disease by analysis of the LC-MS profile. These results were consistent with the study of Zemdegs et al. [71], where metformin treatment (300 mg/kg/day) reduced circulating BCAA levels in insulin-resistant mice.

The reason why those responses from humans and mice were different is unclear, but it might be related to the difference in metformin transportation by organic cation transporters (OCTs), which play a role in the hepatic and renal transport of metformin, and their distribution varies immensely among species [13,14,72]. Additionally, the much higher dose of metformin treatment in animal models than in humans could also make it difficult to translate the results from rodents to humans.

The plasma metabolome from metformin treatment reveals the relevance between metformin and purine metabolism. According to Jenkins et al. [73], several intermediates of purine breakdown were significantly elevated in T2DM mice with metformin treatment. After five weeks of the treatment, xanthosine, inosine, and urate significantly increased in a dose-dependent manner, but candidate tissues that are primarily responsible for the systemic changes were not specified. On the other hand, the significant decrease in inosine and hypoxanthine in serum samples were reported from non-diabetes mice treated with metformin [61].

## 4. Urinary Metabolome and Metformin Treatment

Urine is the most commonly used specimen in metabolomics with its ease of collection and sampling [74]. It is plausible that metabolites in the urine are end products of the biological system and are linked to biological phenotypes. Identification, quantification, and following analysis of urinary metabolites have been studied for diagnosis, health monitoring, and drug metabolism [75]. Urine sampling is non-invasive and requires less pre-treatment than other biofluids due to its low protein content and less chemical complexity. However, sample collection should be performed in a consistent manner because the individual metabolite pattern in urine can be highly affected by various pre-analytical factors, including day and time of collection, physical activity, fasting/feeding, and many other factors [76]. According to the HMDB database, over 2600 metabolites have been identified or quantified from urine, and new biomarkers or metabolites have been discovered by the improvement in analytical technology.

### 4.1. Effect of Metformin Treatment in Insuline-Resistant In Vivo Models

According to Pelantová et al. [77], urinary metabolomic profiles in mice with obesity and T2DM changed after treatment with metformin (250 mg/kg daily), in which significant changes in several amino and aliphatic acid derivatives were detected by analysis of ^1^H, ^1^H-^1^H COSY, and ^1^H-^13^C HSQC NMR experiments. Among the metabolites, *N*-carbamoyl-β-alanine level was significantly decreased by metformin and its decrease indicated the efficacy of the metformin therapy in an oral glucose tolerance test (OGTT). Dong et al. [78] carried out a urinary metabolomic profiling study in Zucker diabetic fatty (ZDF) rats in response to metformin (200 mg/kg/day), glimepiride (5 mg/kg/day), and their combination using LC-MS analysis. From partial least squares discriminant analysis (PLS-DA) of urine metabolic profiles between control, ZDF model, and ZDF-metformin groups, three metabolites, including citric acid, sphingosine, and succinoadenosine, were found significantly reverted to normal levels after metformin therapy. Pan et al. [79] reported the LC-MS-based urinary metabolomics study of the hypoglycemic decoction and metformin (100 mg/kg/day) in a T2DM rat model, revealing the reduction in tricarboxylic acid (TCA) cycle-associated metabolites, including citrate, isocitrate, and cortisol, and the elevation of phenylalanine and 1-methylhistamine. The other urinary metabolomics research reported similar results, where metformin treatment significantly influenced the suppression of TCA cycle metabolism. According to the NMR-based metabolomics study of Maulidiani et al. [80], four metabolites, citrate, alpha-ketoglutarate, succinate, and fumarate, decreased in T2DM rats, and their concentration levels were further reduced after metformin treatment (300 mg/kg/day), which meant metformin suppressed the TCA cycle. Zhu et al. [81] reported the significant reduction in pantothenic acid and malic acid in metformin-treated mice (250 mg/kg/day) by using LC-MS analysis, meaning the suppression of the TCA cycle. Metformin treatment also improved the glucose (decreasing D-glucose) and nucleotide metabolism (decreasing 1-methylnicotinamide). Gut microbiota-produced metabolites including trimethylamine (TMA), phenylacetylglycine (PAG) and indoxyl sulfate levels were observed in the urine, revealing the increased growth or activity of gut microbiota by metformin treatment. Mediani et al. [82] performed NMR-based untargeted metabolomic analysis to understand the biochemical changes in streptozotocin (STZ)-induced, normal-, and obese-diabetic rates with metformin treatment (150 mg/kg/day), suggesting that metformin may provide better improvement for T2DM complications and insulin sensitivity for obese diabetes rather than normal diabetes. Lee et al. [83] also carried out an LC-MS-based untargeted urinary metabolomics study in diabetes and prediabetes rat models, revealing the alteration of urine metabolomes. For prediabetic rats, metformin treatment (250 mg/kg/day) altered the urinary metabolite profile to be more like that of healthy rats (Table 3).

### 4.2. Effect of Metformin Treatment on Human Urine Metabolome

Cho et al. [84] studied the metabolomic changes in 14 healthy volunteers in response to single-dose metformin treatment (1000 mg). In particular, four metabolites, including cortisol, retinyl β-glucuronide, betaine, and cholic acid glucuronide, were identified and quantified by LC-MS analysis in this study. Among them, cortisol and its metabolite hydroxycortisol were significantly decreased after metformin administration. According to Park et al. [85], the response to metformin in early-phase T2DM patients could be predicted by their urine metabolomic profiles acquired from GC-MS, which showed significantly different metabolites as markers of metformin response. In particular, citric acid and hippuric acid were higher and myoinositol was lower in the metformin responder group than the non-responder group. These results suggested that metabolic differences enabled them to discriminate between the metformin responder and non-responder groups efficiently.

## 5. Cell and Tissue Metabolome and Metformin Treatment

Global metabolomics analyses using body fluid provide valuable results for the understanding and prediction of disease. Nevertheless, the mechanism of a disease or the drug response is often tissue- or cell-based and it is advantageous to analyze metabolomic changes directly in the tissue or cells [86]. The cell is the basic structural, functional, and biological unit of all known organisms, and tissue is a cellular organizational level between cells and organisms. Therefore, the cell and tissue metabolome can be defined as the set of all the metabolites present in them, and it is plausible that those metabolites are considered as the basic indicators of cell and tissue phenotypes [87]. Thus, metabolomics is a promising approach to explore those phenotypes and is useful in medical and life science research to understand the metabolism or mechanism of pathology or drug response. Cell-based study is widely used in disease research to investigate the molecular mechanism of the progression, response, and therapeutic resistance in disease status. Additionally, a cell-based approach is less expensive, and it is easier to control the environment and interpret the results, than analysis of animal models or human subjects. The comprehensive analysis of the metabolic alterations in the metabolite levels in cells can provide information as to the underlying causes of disease and targets for pharmacological intervention [35]. Initially, when the metabolic changes take place in response to a disease or drug response, tissue analyses are necessary. Unlike biofluids such as plasma and urine, cell and tissue metabolites are not diluted and enable us to provide stronger biomarkers [88].

The antineoplastic activity of metformin has been confirmed against several cancer types in vitro and in vivo [89]. However, the underlying mechanisms of metformin are not fully understood, and several studies have tried to apply metabolomics to explain the anticancer properties of metformin. According to Janzer et al. [90], biguanides including metformin and phenformin decrease TCA cycle intermediates including citrate, aconitate, isocitrate, alpha-ketoglutarate, fumarate, malate, and glutamate in cancer cell lines by using LC-MS. These results provided pharmacological and pathological evidence that mitochondrial complex I is a target of anticancer properties of biguanides. Soliman et al. [91] investigated the metabolic effect of metformin in the pancreatic cancer cell line using LC-MS, revealing the significant decrease in TCA cycle intermediates (citrate, isocitrate, and malate) which made a synergy with mTOR complex inhibition for reduced energy production. Zhang et al. [92] performed NMR-based metabolomic analysis of metformin-induced anticancer effects and demonstrated that metformin treatment could inhibit proliferations of human cholangiocarcinoma (CCA) cells, observing the remarkable decrease in glucose, fumarate, and alanine and increase in NAD^+^, UDP-GlcNAc, and BCAAs, implying the occurrence of autophagy and cell-cycle arrest. Liu et al. [93] carried out an LC-MS-based integrative metabolomics analysis of metformin action in ovarian cancer and observed the altered mitochondrial metabolism in human tumors from patients taking metformin with the suppression of TCA intermediates and short chain acyl carnitines. Those results consistently indicated that metformin affected the TCA cycle, which is the main energy production metabolism for cells.

Yan et al. [94] performed integrative metabolomics analysis to systematically examine the effect of metformin on metabolic reprogramming, revealing the AMPKα-independent metabolic alteration in mouse embryonic fibroblast (MEF) cells. Notably, metformin treatment significantly reduced the levels of citrate and succinate and elevated the cellular lactate level, which was evidence of TCA cycle suppression. Amino acid levels were also altered on the increase in serine, hypotaurine, and 3-methyl-2-oxovaleric acids and the decrease in proline, 3-guanidinobutyric acid, and aspartate. LV et al. [95] observed 28 metabolites in insulin-resistant cells by analyzing ^1^H NMR spectra and reported increased levels of valine, leucine, isoleucine, threonine, lactate, glutamate, glutamine, creatine, choline, glycine, and carnosine, and decreased level of aspartate, *O*-phosphocholine, taurine, ATP, and UDP-GlcNAc in metformin treatment. In detail, low metformin treatment rectified glucose metabolic imbalance and regulated oxidative stress and phospholipid and energy metabolism. A high dose of metformin induced apoptosis and inhibited tumor cell growth through energy metabolism, phospholipid metabolism, and glucose catabolism.

Riera-Borrull et al. [96] performed a targeted metabolomic study to investigate the protective effect of metformin against metabolic disturbances provoked by a high-fat diet (HFD) in a mice model by comparing the metabolic alteration on adipose tissue and liver in response to metformin treatment. In white adipose tissue (WAT), metformin treatment led to a significant decrease in the levels of the BCAAs and increased glycolytic intermediates (phosphoenolpyruvate, pyruvate, and lactate) and TCA cycle intermediates. On the other hand, phosphoenolpyruvate levels significantly decreased in liver tissue. Hao et al. [97] investigated metabolism disturbances induced by corticosterone (CORT) and determined that metformin can reverse these effects. In their metabolomic approach, metformin treatment altered several metabolites involved in the pathway of the TCA cycle (isocitrate, citrate, alpha-ketoglutarate, oxaloacetate, malic acid, succinate, and fumarate) and the pathway of glycolysis and gluconeogenesis (3-phospho-D-glycerate, fructose 6-phosphate, glucose 6-phosphate, and pyruvate) in liver tissue. CORT-induced depression-like behaviors were also attenuated by metformin treatment.

According to Li et al. [98], metformin improved locomotor function, but attenuated cognitive function in normoglycemic male mice. Brain metabolisms in normoglycemic mice are altered with the elevation of dimethylglycine, histidine, and choline and the reduction in malic acid, thymidine, dihydroxyacetone-phosphate (DHAP), and myo-inositol, revealing the inhibition of mitochondrial glycerol-3-phosphate dehydrogenase (mGPDH), reduced oxidative phosphorylation, and increased glycolysis.

## 6. Conclusions

**Figure 3:**
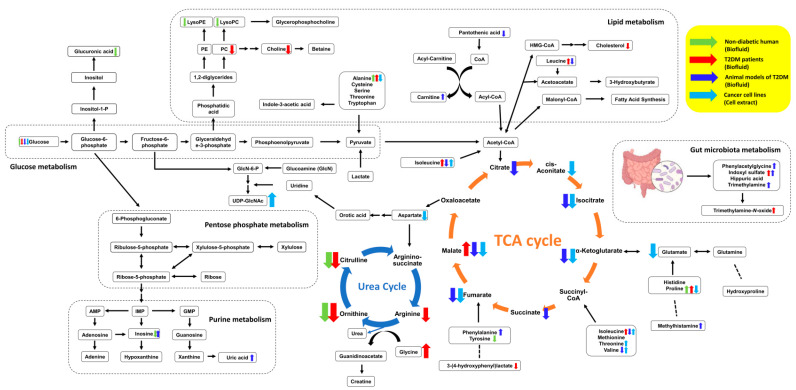
Pathways and metabolites significantly affected by metformin treatment in non-diabetic human (biofluid, green), T2DM patients (biofluid, red), T2DM animal models (biofluid, blue), and cancer cells (cell extract, sky blue).

Metformin is widely studied for its therapeutic potentials as well as the treatment of type 2 diabetes mellitus (T2DM). The metabolic profiles associated with various metabolisms and pathways were significantly influenced by metformin treatment, regardless of metabolic condition, organism, or biospecimen, but the changes were not identical to each other. As shown in Figure 3, metformin treatment is associated with TCA cycle, urea cycle, glucose metabolism, lipid metabolism, or gut microbiota metabolism, but metabolic changes differed by organism, condition, and biospecimen. For example, the changes in BCAA levels were different in T2DM patients and animal models. Metabolomic research with metformin treatment rose in the early 2010s and has increased during the last 10 years, but the number of publications, especially based on clinical research, is still not enough to obtain robust results. Nevertheless, the observation of metabolic changes and the effort to explain those alterations are anticipated to be helpful to understand a mechanism of metformin and provide a better therapeutic usage of metformin.

## Figures and Tables

**Table 1:** Summary of plasma metabolite alteration following metformin treatment in non-diabetic participants (*p* ≤ 0.05) ^a^.

Name	Change	Method	Metabolic Pathway	Refs.
Alanine	up	LC-MS, NMR	Alanine and aspartate metabolism	[24,55,59]
Glucuronic acid	down	LC-MS	Amino sugar metabolism	[24,55,61]
3-Hydroxymethylglutaric acid	up	LC-MS	BCAA metabolism	[24,55]
Hippuric acid	up or down ^b^	LC-MS	Benzoate metabolism Gut microbiota metabolism	[24,61]
Linoleyl Carnitine	down	LC-MS	Fatty acid metabolism (Acyl carnitine)	[24,55]
gamma-Glutamylleucine	up	LC-MS	Gamma-glutamyl amino acid metabolism	[24,55]
Glucose	up	LC-MS	Glycolysis, gluconeogenesis and pyruvate metabolism	[24,55]
Aminoadipic acid	up	LC-MS	Lysine metabolism	[24,55]
2-Ketobutyric acid	up	LC-MS	Methionine, cysteine, SAM and taurine metabolism	[24,55]
Phenylacetate	up	LC-MS	Phenylalanine metabolism	[24,55]
16:0 LPC	down	LC-MS	Phosphatidylcholine metabolism	[24,54,55]
18:0 LPC	down	LC-MS	Phosphatidylcholine metabolism	[24,54,55]
Arachidonic acid	up	LC-MS	Polyunsaturated fatty acid metabolism	[24,61]
Cholic acid	down	LC-MS	Primary bile acid metabolism	[24,55]
Hypoxanthine	up or down ^b^	LC-MS	Purine metabolism	[24,61]
Inosine	down	LC-MS	Purine metabolism	[24,61]
Sphinganine 1-phosphate	down	LC-MS	Sphingolipid synthesis	[24,55]
Dopamine3-O-sulfate	up	LC-MS	Tyrosine metabolism	[24,55,61]
Tyrosine	down	LC-MS, NMR	Tyrosine metabolism	[59,60,61]
Arginine	up or down ^b^	LC-MS	Urea cycle	[24,55]
Citrulline	down	LC-MS	Urea cycle	[24,55]
Ornithine	down	LC-MS	Urea cycle	[24,61]
Proline	up	LC-MS	Urea cycle	[24,55]

^a^ The metabolites altered in multiple studies with *p*-value ≤ 0.05 are listed on the table; ^b^ Opposite results were reported in the literature.

**Table 2:** Summary of plasma metabolite changes following metformin treatment in T2DM patients (*p* ≤ 0.05) ^a^.

Name	Change	Method	Metabolic Pathway	Refs.
Alanine	up	LC-MS, NMR	Alanine and aspartate metabolism	[24,65,67]
Isoleucine	up	LC-MS, NMR	BCAA metabolism	[60,67,68]
Leucine	up	LC-MS	BCAA metabolism	[24,60,68]
gamma-glutamyltyrosine	down	LC-MS	Gamma-glutamyl amino acid	[24,64]
Glycine	up	LC-MS, NMR	Glycine, serine and threonine metabolism	[24,67]
Glutaroyl carnitine	down	LC-MS	Lysine metabolism	[24,64]
Phenylalanine	up	LC-MS	Phenylalanine metabolism	[24,62]
PC ae C36:4 ^c^	down	LC-MS	Phosphatidylcholine metabolism	[63,65]
PC ae C38:5 ^c^	down	LC-MS	Phosphatidylcholine metabolism	[63,65]
PC ae C38:6 ^c^	down	LC-MS	Phosphatidylcholine metabolism	[63,65]
Choline	down	LC-MS	Phospholipid metabolism	[24,64]
Trimethylamine-*N*-oxide	up	LC-MS, NMR	Phospholipid metabolism Gut microbiota metabolism	[24,62]
Inosine	up or down ^b^	LC-MS	Purine metabolism	[24,64]
Cholesterol	down	LC-MS	Sterol metabolism	[24,64]
Malate	up	LC-MS	TCA Cycle	[24,64]
3-Indoxyl sulfate	up	LC-MS	Tryptophan metabolism Gut microbiota metabolism	[24,64]
Tryptophan	up or down ^b^	LC-MS	Tryptophan metabolism	[24,62]
3-(4-hydroxyphenyl)lactate	down	LC-MS	Tyrosine metabolism	[24,64]
Arginine	down	LC-MS	Urea cycle	[24,64]
Citrulline	down	LC-MS	Urea cycle	[24,64,65]
Ornithine	down	LC-MS	Urea cycle	[24,64]
Proline	up	LC-MS	Urea cycle	[24,65]

^a^ The metabolites altered in multiple studies with *p*-value ≤ 0.05 are listed on the table; ^b^ Opposite results were reported from the literature. ^c^ PC ae: phosphatidylcholine acyl-alkyl.

**Table 3:** Summary of metabolite changes following metformin administration in insulin-resistant in vivo models. (*p* ≤ 0.05).

No.	Name	Changes	Biospecimen	Animal	Method	Metabolic Pathway	Refs.
1	1-Methylhistamine	up	Urine	Rat	LC-MS	Histidine metabolism	[79]
2	alpha-ketoglutaric acid	down	Urine	Rat	NMR	TCA cycle	[80]
3	Carnitine	up	Blood	Mice	LC-MS	Lipid metabolism	[69]
4	Citric acid	down	Urine	Rat	LC-MS, NMR	TCA cycle	[78,79,80]
5	Cortisol	down	Urine	Rat	LC-MS	Lipid metabolism	[79]
6	Fumarate	down	Urine	Rat	NMR	TCA cycle	[80]
7	Isocitric acid	down	Urine	Rat	LC-MS	TCA cycle	[79]
8	Isoleucine	down	Blood	Mice	LC-MS	BCAA metabolism	[69,70,71]
9	Leucine	down	Blood	Mice	LC-MS	BCAA metabolism	[70,71]
10	Malic acid	down	Urine	Mice	LC-MS	TCA cycle	[81]
11	N-carbamoyl-β-alanine	down	Urine	Rat	NMR	Uracil metabolism	[77,78]
12	Pantothenic acid	down	Urine	Mice	LC-MS	CoA biosynthesis	[81]
13	Phenylalanine	up	Urine	Rat	LC-MS	Phenylalanine, tyrosine metabolism	[79]
14	Sphingosine	down	Urine	Rat	LC-MS	Sphinganine metabolsim	[78]
15	Succinate	down	Urine	Rat	NMR	TCA cycle	[80]
16	Succinoadenosine	up	Urine	Rat	LC-MS	Purine metabolism	[78]
17	Valine	down	Blood	Mice	LC-MS	BCAA metabolism	[70,71]
18	Trimethylamine	up	Urine	Mice	LC-MS	Gut microbiota metabolism	[81]
19	Phenylacetylglycine	up	Urine	Mice	LC-MS	Gut microbiota metabolism	[81]
20	Indoxyl sulfate	up	Urine	Mice	LC-MS	Gut microbiota metabolism	[81]
